# Research note: Comparative identification of higher-order quadruplex structures in the *LEP* locus of galliformes

**DOI:** 10.1016/j.psj.2026.106985

**Published:** 2026-04-19

**Authors:** Jinsoo Ahn, Kichoon Lee

**Affiliations:** Department of Animal Sciences, The Ohio State University, Columbus, OH 43210, USA

**Keywords:** G-quadruplex, i-motif, *LEP*, Higher-order quadruplex, Galliformes

## Abstract

Four-stranded nucleic acid secondary structures known as quadruplexes, specifically G-quadruplexes (G4s) and i-motifs (iMs), form in G-rich and C-rich sequences, respectively. Although these quadruplex structures have been identified within regulatory regions and are involved in gene regulation, their distribution within the GC-rich avian *LEP* locus remains to be elucidated. Leptin (LEP) influences growth and nutrient utilization in vertebrates by regulating appetite; however, the regulatory mechanisms governing *LEP* expression in chickens are still poorly understood despite the importance of production efficiency in poultry systems. The objectives of this study were to characterize the GC-rich chicken *LEP* locus and perform comparative analyses of the avian *LEP* genomic landscape. Following the identification of *LEP*-containing chicken genomic sequences via the Basic Local Alignment Search Tool (BLAST), we employed approaches to identify G4- and iM-based potential higher-order quadruplex sequences (PHOQSs). Consequently, 23 G4-based PHOQSs and nine iM-based PHOQSs were identified in the Silkie chicken *LEP* locus. Moreover, G4 and iM configurations within these PHOQS structures were predicted, indicating distinct spatial arrangements between the two structural elements (G4s and iMs). Our comparative analyses revealed that these higher-order quadruplexes are conserved across chicken breeds and appear to be conserved in the quail *LEP* locus, whereas such conservation was absent in the mallard duck. These findings suggest a unique gene regulatory mechanism in the chicken and quail *LEP* loci, which appears distinct from that of the mallard duck and may contribute to the leptin biology within Galliformes.

## Introduction

G-quadruplexes (G4s) are four-stranded nucleic acid secondary structures formed on G-rich strands, in which guanines are mutually bonded by Hoogsteen hydrogen base pairing to form a planar G-tetrad (or G-quartet) (Varshney et al., 2020). These tetrads stack upon one another and are stabilized by a central monovalent cation, notably potassium (K^+^). The folding topology of intramolecular G4s is classified into parallel, antiparallel, and hybrid types, according to the orientation of the strands (Liew et al., 2024). On the other hand, intercalated motifs (i-motifs; iMs) are four-stranded nucleic acid secondary structures formed in C-rich sequences, where two parallel-stranded duplexes are held together in an antiparallel orientation by intercalated, hemiprotonated cytosine–cytosine (C:C^+^) base pairs (Abou Assi et al., 2018).

Additionally, the simultaneous formation of i-motif and G4 structures on the same strand has been reported under acidic conditions favoring i-motif stability in the presence of a G4-compatible cation (Zhou et al., 2013). The close proximity of C-rich and G-rich regions may also result in an intramolecular hairpin duplex referred to as quadruplex–hairpin conversion (Qiu et al., 2015). Beyond these structural conformations, parallel-stranded DNA G-quadruplexes have been shown to form within promoter regions, suggesting a key role in transcriptional regulation (Agrawal et al., 2013). Furthermore, the distribution of potential higher-order quadruplex sequences (PHOQSs) across various chromosomes in the human genome has been documented, highlighting their widespread occurrence (Belmonte-Reche and Morales, 2020).

The chicken *LEP* gene is characterized by an extremely high GC content (approximately 70% in the coding sequence) and a markedly low level of expression, especially in adipose tissue, unlike in mammals (Seroussi et al., 2016). This unique sequence composition and expression deficiency, along with its long-standing absence from both current and previous chicken reference genomes, led to the hypothesis of a distinctive regulatory mechanism for chicken *LEP*. In a broader genomic context, previous studies have identified G-quadruplexes within transcriptional regulatory regions across the chicken genome (Abe and Gemmell, 2014), although omitting the *LEP* locus due to its absence from the reference genomes.

Here, following the identification of *LEP*-containing chicken genomic sequences via BLAST, we report the identification of highly dense, higher-order DNA quadruplex structures within the *LEP* locus of the chicken and their conservation across chicken breeds and quail. In chickens, we observed that G4-based higher-order structures exhibit a more compact spatial arrangement compared to iM-based structures, a pattern that remained consistent across analyzed chicken breeds. Comparative analyses revealed that these higher-order structures are highly conserved across multiple chicken breeds, with similar patterns observed in the quail; however, they are notably absent in the mallard duck. Taken together, these findings highlight a highly enriched landscape of higher-order quadruplex structures and suggest a novel mechanism for gene regulation within the *LEP* locus, specifically across galliform species, including chickens and quail.

## Materials and methods

### Blast search

Given the absence of the *LEP* gene in the current and previous annotation releases of the chicken reference genomes (GRCg7b from the Broiler haplotype and GRCg6a from Red Jungle Fowl, respectively), as well as in the alternative GRCg7w assembly (White Leghorn haplotype), BLAST searches were performed against other genome assemblies. Specifically, chicken genome assemblies deposited in the NCBI Genome database were queried using BLASTn with the published chicken *LEP* coding sequence (GenBank: KT970642.1). In contrast, the duck *LEP* gene is annotated in the reference genome (IASCAAS_PekinDuck_T2T). For the quail, a BLASTn search using the published *LEP* sequence (GenBank: MK689854.1) was conducted against the reference genome (Coturnix japonica 2.1) to identify the target locus.

### Genome acquisition and sequence extraction

For chicken (*Gallus gallus*), genome assemblies containing the *LEP* gene were obtained from Silkie (CAU_Silkie_2.0), Huxu (GGswu), Wenchang (ASM4043665v1), Game fowl (ASM4192031v1), and an Indonesian ecotype (ASM4646395v1). These assemblies represent the currently available chicken genome sequences containing the *LEP* locus in the NCBI Genome database, and assemblies from other breeds, including industrial commercial breeds, lack coverage for this region. These genome assemblies were used to extract the genomic sequences encompassing the *LEP* gene. For duck (mallard; *Anas platyrhynchos*), the reference genome IASCAAS_PekinDuck_T2T (GCF_047663525.1) was retrieved to extract the genomic sequence spanning the *LEP* gene. For quail (Japanese quail; *Coturnix japonica*), the reference genome Coturnix japonica 2.1 (GCF_001577835.2) was retrieved to extract the genomic sequence comprising a *LEP* exon and a partial intron.

### Identification of G4-based higher-order structures and individual G4s

The R package G4-iM Grinder was employed to identify G4-based potential higher-order quadruplex sequences (PHOQSs) using the M3A method (a non-overlapping, size-unrestricted approach for detecting higher-order structures) with default parameters (Belmonte-Reche and Morales, 2020). These parameters include: *RunComposition* = "G"; *MinNRuns* = 4 (requiring at least four G-runs to support the formation of an intramolecular G4 structure); *MinRunSize* = 3 (minimum of three guanines per G-run); and *BulgeSize* = 1 (allowing a maximum of one non-guanine nucleotide within a G-run). G4-based PHOQSs with a high probability of structural formation [score ≥ 40, a threshold recommended by G4-iM Grinder and verified against known G4s (Belmonte-Reche and Morales, 2020)] were identified on the postive strand. Subsequently, the GiG.M3Structure function was used to analyze all potential quadruplex sequence (PQS) arrangements within the PHOQSs through 10,000 iterations of random assignment. The most probable non-overlapping configurations of multiple G4 structures were determined by identifying the best-scoring Random Allocation normalized High-scoring (RAnH) conformation within each PHOQS, from which the final G4 sequences were extracted.

### Identification of iM-based higher-order structures and individual i-motifs

G4-iM Grinder was employed to detect iM-based PHOQSs using the M3A method with the parameter *RunComposition* = "C" (Belmonte-Reche and Morales, 2020). Other search and optimization parameters, including the threshold for structural formation (applying a corresponding threshold of score ≤ −40 to account for the inverse scoring scale), were maintained as described above for the G4-based PHOQSs. Accordingly, the GiG.M3Structure function was utilized to analyze all C-based potential i-motif sequences (PiMS), and the best-scoring RAnH conformation were identified to extract the final iM sequences.

### Folding topology prediction

To determine the structural orientation of the identified G4 sequences, the machine-learning-based G4ShapePredictor (G4SP) was used to predict folding topologies, including parallel (4 + 0), antiparallel (2 + 2), or hybrid (3 + 1) configurations, with the default RandomForest model (Liew et al., 2024).

## Results and discussion

### Identification of higher-order quadruplex structures in the *LEP* locus of chicken

**Fig. 1:**
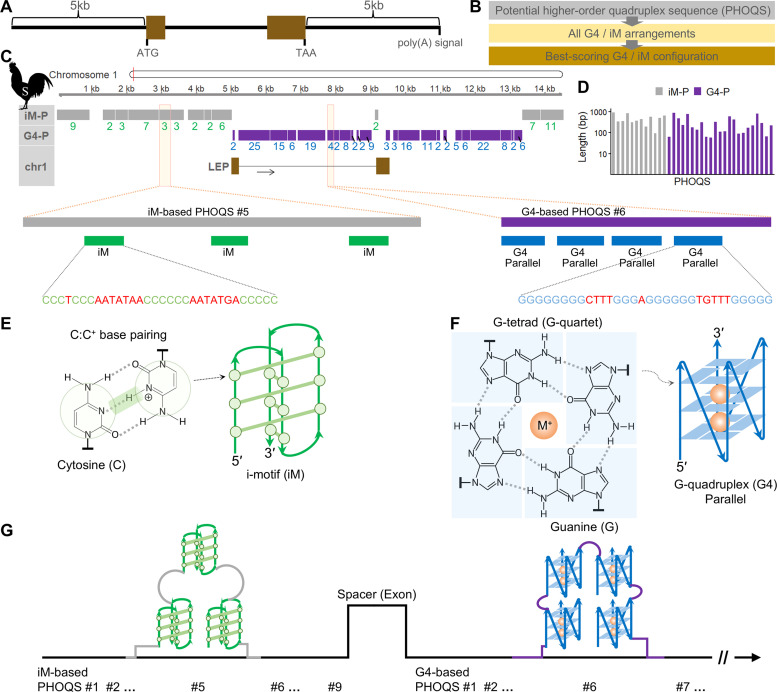
Genomic distribution and organization of DNA quadruplex structures. (A) Analyzed chicken LEP locus encompassing the coding sequence (NCBI GenBank: KT970642.1). (B) Workflow for the identification of higher-order assemblies and quadruplex configurations. (C) PHOQSs in genomic DNA (plus strand) from the Silkie (S) chicken breed. iM-P, iM-based PHOQS; G4-P, G4-based PHOQS. Numbers indicate the count of specific iMs or G4s predicted to have the best-scoring configurations within each PHOQS. In sequence snippets, G/C-runs are shown in blue/green, while loop residues are highlighted in red. (D) Log_10_-transformed lengths (bp). (E) Hemiprotonated cytosine–cytosine (C:C^+^) base pairing and the intercalated motif (i-motif; iM). (F) Hoogsteen hydrogen bonding forming a planar G-tetrad (G-quartet) stabilized by a monovalent cation (M^+^), and the G-quadruplex (G4) structure with parallel conformation. (G) Schematic of potential stabilizing arrangements for iMs and G4s within the exemplified PHOQSs.

To investigate the chicken *LEP* genomic locus, we first conducted BLAST searches and identified approximately 98% sequence identity on chromosome 1 of the latest Silkie chicken genome assembly (CAU_Silkie_2.0). Unlike chicken reference genomes in which *LEP* is absent from chromosome 1, its presence in this assembly is likely due to the high contiguity of this recently generated genome assembly. Subsequently, we extracted the genome sequence spanning 5 kb upstream of the start codon and 5 kb downstream of the stop codon, using the published *LEP* coding sequence (GenBank: KT970642.1) as a reference (Fig. 1A). For the extracted Silkie genomic sequence, the identification of PHOQSs and their constituent PQS arrangements (representing all i-motif and G4 arrangements) was performed, followed by the determination of the best-scoring RAnH-based iM and G4 configuration within each PHOQS (Fig. 1B).

As a result, we identified 23 G4-based PHOQSs, primarily within the intron and downstream of the stop codon, with each PHOQS containing multiple constituent G4s (Fig. 1C). Additionally, nine iM-based PHOQSs were identified in the region upstream of the start codon, while three others were found at approximately 9 kb and between 13.3–14.5 kb, with each PHOQS comprising multiple iMs (Fig. 1C). In addition to the concentrated higher-order structures, we identified a total of 210 individual G4s. This G4 density is remarkably high, considering that previous studies reported hundreds to thousands of potential G-quadruplexes in gene regulatory regions across the entire chicken reference genome (Abe and Gemmell, 2014).

### Characterization of G4 and i-motif-based higher-order quadruplex structures

Comparison of the exemplified G4-based PHOQS #6 and iM-based PHOQS #5 indicated a more compact arrangement of G4s compared to the broader spacing of i-motifs within their respective PHOQSs (Fig. 1C). The length of G4-based PHOQSs ranged from 60 to 891 bp, with the longest (891 bp) containing 25 constituent G4s; meanwhile, iM-based PHOQSs ranged from 97 to 930 bp, with the longest (930 bp) comprising 9 iMs (Fig. 1D). Taken together, these observations on quadruplex motif spacing and frequency suggest that substantially more G4s than iMs are typically required to construct similar-sized PHOQSs.

While Hoogsteen hydrogen bonding can support parallel, antiparallel, and hybrid topologies (Liew et al., 2024), as exemplified by the parallel G4s within G4-based PHOQS #6 (Fig. 1C), the C:C^+^ base pairing is associated with an antiparallel conformation in the context of the i-motif (Fig. 1E,F). Moreover, the stable conformations of G4s and iMs within PHOQSs may be required to maintain structural integrity of the PHOQS clusters (Fig. 1G). In addition to the reported co-occurrence of G4s and iMs on the same strand separated by spacer sequences (Qiu et al., 2015), the G4-based PHOQSs and iMs-based PHOQSs identified here on the same strand appear to flank a spacer exon, as a structural gap was observed across the exonic region (Fig. 1G). This is consistent with the lower incidence of quadruplexes observed in transcribed regions (Huppert and Balasubramanian, 2005).

### Conservation of higher-order quadruplex structures within the LEP locus of chicken and quail

**Fig. 2:**
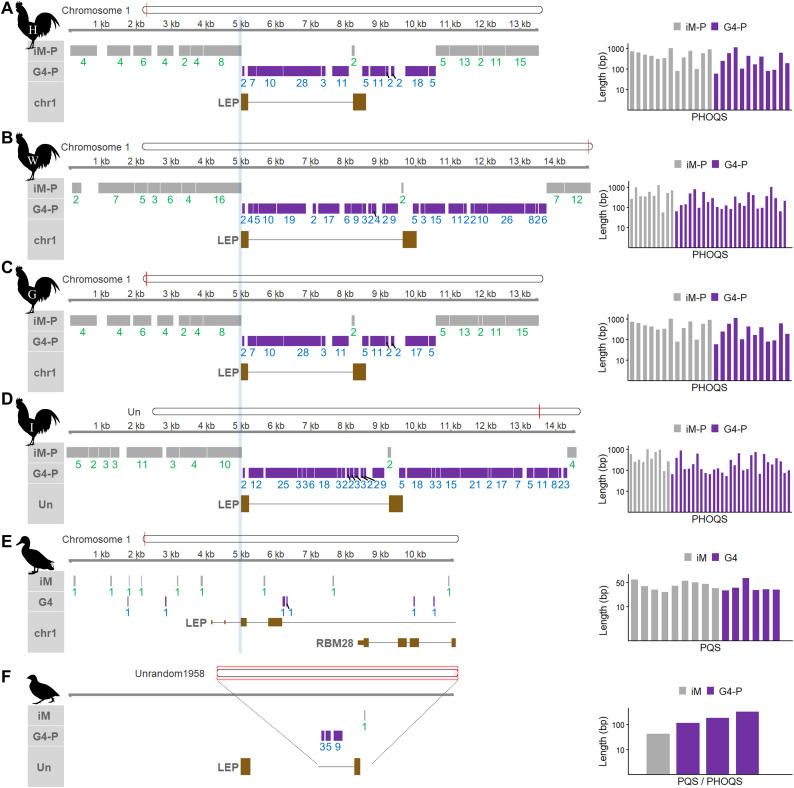
Comparative genomic distribution of DNA quadruplex structures across avian species. (A–D) PHOQS distribution on the plus strand of the LEP locus in four chicken breeds: (A) Huxu (H), (B) Wenchang (W), (C) Game fowl (G), and (D) Indonesian ecotype (I). The LEP gene is located on chromosome 1 (red bars in ideograms), except for the Indonesian ecotype, where it is situated on an unplaced scaffold (JBBHEU010000013.1). (E) Distribution of isolated G4s and i-motifs (PQSs; labeled 1) in the mallard duck. Note the absence of PHOQS clusters. (F) G4-based PHOQSs in the quail unplaced scaffold (Unrandom1958; NW_015440728.1; 1,649 bp) and a single isolated i-motif. The first exon is derived from the quail LEP coding sequence (GenBank: MK689854.1). An assembly gap within the intron is indicated by a blank space. Un, Unplaced scaffold; iM, single i-motif; G4, single G-quadruplex.

Comparative analyses of higher-order quadruplex structures were conducted across avian species, including four additional chicken breeds, mallard duck, and quail (Fig. 2). Our analysis revealed that the chicken *LEP* intron harbors dense clusters of G4-based PHOQSs across all four additional breeds, including Huxu, Wenchang, Game fowl, and an Indonesian ecotype (Fig. 2A–D). In the region downstream of the stop codon, this dense pattern was maintained in Wenchang and the Indonesian ecotype (Fig. 2B,D); however, the PHOQS clusters in Huxu and Game fowl were shorter, reflecting the reduced genomic sequence length in these two breeds (Fig. 2A,C). Notably, the G4- and iM-based PHOQS distribution and sequence length in the Silkie chicken (Fig. 1C) were more similar to those observed in the Wenchang and Indonesian ecotype (Fig. 2B,D). In contrast, in the mallard duck, G4- and iM-based PHOQSs characterized by multiple quadruplexes were absent, and only isolated, single G4s and iMs (PQSs) with substantially shorter lengths were detected (Fig. 2E). On the other hand, in the quail, despite the limited length of the analyzed unplaced scaffold (Unrandom1958; 1,649 bp), which did not cover the entire *LEP* locus, three G4-based PHOQSs were identified alongside a single isolated i-motif (Fig. 2F).

Although the *LEP* gene in the Wenchang chicken is atypically located in a subtelomeric region of the q-arm on chromosome 1 rather than the p-arm as observed in other breeds, and while future sequencing efforts are expected to provide more complete chicken and quail genome assemblies, our current results suggest that dense PHOQS clustering is a characteristic feature of galliform species (including chickens and quail). In contrast, this clustering is entirely absent in the mallard duck, an anseriform species. While PHOQS distribution across various chromosomes in the human genome has been previously reported (Belmonte-Reche and Morales, 2020), the highly condensed PHOQS clusters identified in this study within the chicken *LEP* locus are notable for their exceptional density. Previous studies have shown that *LEP* expression in chickens is detected in multiple tissues, including the brain, pituitary gland, pancreas, and liver (Seroussi et al., 2016). This broad but low-level expression pattern, with differential levels among tissues, suggests the involvement of regulatory elements controlling tissue-specific transcription. The dense PHOQS clusters identified in the *LEP* locus may therefore function as structural regulatory elements influencing chromatin accessibility and gene expression. Consequently, further research is required to investigate the stabilization of these structures using small molecule ligands (Varshney et al., 2020), to further delineate their functional impact on *LEP* expression. In conclusion, these findings indicate that higher-order quadruplex structures are highly conserved within the *LEP* locus across the galliform lineage, as evidenced by their consistent presence in diverse chicken breeds and the quail.

## CRediT authorship contribution statement

**Jinsoo Ahn:** Conceptualization, Data curation, Formal analysis, Investigation, Methodology, Software, Validation, Visualization, Writing – original draft, Writing – review & editing. **Kichoon Lee:** Conceptualization, Funding acquisition, Investigation, Resources, Supervision, Validation, Writing – original draft, Writing – review & editing.

## Disclosures

The authors declare that they have no known competing financial interests or personal relationships that could have appeared to influence the work reported in this paper.
